# Supplementary material for: Whole-Genome Differentially Hydroxymethylated DNA Regions among Twins Discordant for Cardiovascular Death
Source: Genes (Basel). 2021 Jul 29;12(8):1183. doi: 10.3390/genes12081183 (PMC8392630; doi:10.3390/genes12081183)
Supplement: Supplementary file 1 [file genes-12-01183-s001.zip › genes-1314486-supplementary/genes-1314486 - supplementary files/Table S1-S2-S4_Figure S1-S3_SuppInfo.pdf]

Supplementary Table S1. Characteristics at exam 3 (1986-1987) of twin pairs discordant for cardiovascular death[1]

Characteristics	CVD-dMZ (n=19 pairs)				CVD-dDZ (n=19 pairs)			
	Total	Case Twins	Control Twins	P value[2]	Total	Case Twins	Control Twins	P Value[2]
Discordant pairs, n	19	19	19	–	19	19	19	-
Age at exam 3 (y)	65.4 (2.5)	65.4 (2.5)	65.4 (2.5)	1.00	64.9 (3.2)	64.9 (3.2)	64.9 (3.2)	1.00
Age at death (y)	79.0 (7.9)	74.3 (5.7)	83.7 (7.1)	<0.0001	80.6 (7.1)	76.7 (5.2)	84.5 (6.7)	<0.0001
Cigarette smoking, n (%)				0.21				0.96
Never smokers	9 (24)	3 (16)	6 (32)		7 (19)	3 (16)	4 (22)	
Former smokers	24 (63)	13 (68)	11 (58)		23 (62)	11 (58)	12 (67)	
Current smokers	5 (13)	3 (16)	2 (10)		7 (19)	5 (26)	2 (11)	
Education,[3] (y)	13.9 (2.6)	14.2 (3.1)	13.6 (2.2)	0.36	12.2 (3.0)	11.9 (2.8)	12.5 (3.1)	0.23
Body mass index (kg/m^2)	28.2 (3.6)	28.0 (3.4)	28.4 (3.7)	0.39	26.8 (3.8)	26.9 (4.1)	26.7 (3.7)	0.83
Obesity, n (%)	9 (24)	5 (26)	4 (21)	0.66	6 (16)	3 (16)	3 (16)	0.97
Systolic blood pressure (mm Hg)	137 (14)	139 (13)	135 (14)	0.20	136 (12)	138 (14)	134 (8)	0.24
Diastolic blood pressure (mm Hg)	83 (7)	82 (7)	85 (6)	0.13	84 (8)	85 (8)	83 (8)	0.40
Plasma biochemistry								
Total cholesterol (mg/dL)[4]	232 (37)	223 (35)	241 (38)	0.04	214 (39)	215 (35)	213 (43)	0.87
Triglyceride (mg/dL)[5]	163 (75)	172 (90)	153 (56)	0.26	148 (83)	157 (84)	193 (83)	0.35
HDL-C (mg/dL)[4]	43 (11)	42 (11)	44 (10)	0.44	44 (13)	44 (14)	44 (11)	0.86
LDL-C (mg/dL)[4]	157 (33)	146 (29)	166 (36)	0.013	145 (35)	143 (32)	147 (37)	0.69
Postload glucose (mg/dL)[6]	186 (33)	190 (27)	182 (38)	0.50	179 (60)	186 (65)	173 (55)	0.46
Diabetes, n (%)	8 (21)	5 (26)	3 (16)	0.41	9 (24)	6 (32)	3 (16)	0.22
Use of cholesterol-lowering medicine, n (%)	0 (0)	0 (0)	0 (0)	1.00	1 (0.3)	1 (0.5)	0 (0)	1.00
Use of antihypertensives, n (%)	16 (42)	9 (47)	7 (37)	0.35	14 (37)	10 (53)	4 (21)	0.08
Peripheral blood leukocyte composition,[7] %					N/A	N/A	N/A	
CD8+ T-cells	2.6 (3.2)	3.0 (3.7)	2.2 (2.7)	0.44	–	–	–	
CD4+ T-cells	13.9 (5.7)	13.0 (5.4)	14.8 (5.9)	0.11	–	–	–	
Natural killer cells	5.6 (4.1)	5.7 (4.6)	5.6 (3.8)	0.87	–	–	–	
B-cells	4.1 (1.8)	4.1 (2.1)	4.1 (1.6)	0.94	–	–	–	
Monocytes	7.1 (3.2)	7.0 (2.9)	7.1 (3.4)	0.91	–	–	–	
Granulocytes	68.3 (7.9)	68.7 (8.8)	67.9 (7.0)	0.69	–	–	–	

Abbreviations: CVD-dMZ= monozygotic twin pairs discordant for CVD death; CVD-dDZ= dizygotic twin pairs discordant for CVD death; HDL-C=high-density lipoprotein cholesterol; LDL-C= low-density lipoprotein cholesterol

[1]data at exam 3 are presented as mean (SD) unless otherwise specified. Raw values for continuous variables are presented.

[2]case twins versus their co-twin brothers (controls).

[3]from baseline exam (exam 1)

[4]to convert mg/dl of total cholesterol, HDL or LDL cholesterol to mmol/l, divide by 38.67

[5]to convert mg/dl of triglycerides to mmol/l, divided by 88.5.

[6]to convert mg/dl of glucose to mmol/l, divided by 18.02.

[7]estimated from methylation data

Supplementary Table S2. Characterization of differentially hydroxymethylated region among the discovery sample of monozygotic twin pairs discordant for cardiovascular death (N=12 pairs)

	DhMR Features					DhMR Coverage, bp		
	Total DhMRs[4] (n)	Consistent DhMRs[5] (n)	Hyper-DhMRs (n)	Hypo-DhMRs (n)	Size, Mean (range), bp	Total DhMRs[4]	Hyper-DhMRs	Hypo-DhMRs
**Model 1[1]**	16,232	1,893	12,262	3,970	262 (150 -950)	4,264,650	3,209,400	1,055,250
**Model 2[2]**	6,438		5,264	1,174	232 (150-950)	1,497,950	1,229,500	268,450
**Model 3[3]**	7,864		6,411	1,453	228 (150-950)	1,789,550	1,463,950	325,600

Abbreviations: DhMRs=differentially hydroxymethylated regions; hyper-DhMRs = differentially hyper-hydroxymethylated regions (case twins vs. control twins); hypo-DhMRs = differentially hypo-hydroxymethylated regions (case twins vs. control twins)

[1]does not adjust for any covariates while naturally adjust for germline and environmental factors shared between monozygotic co-twins

[2]adjust for blood leukocyte subtypes, including CD4+ T-cells, CD8+ T-cells, natural killer cells, B-cells, and monocytes, in addition to germline and environmental factors shared between monozygotic co-twins

[3]adjust for blood leukocyte subtypes and age, including CD4+ T-cells, CD8+ T-cells, natural killer cells, B-cells, and monocytes, in addition to germline and environmental factors shared between monozygotic co-twins

[4]Benjamini–Hochberg False Discovery Rate (B-H FDR) <0.01

[5]the number of directionally consistent DhMRs among models 1, 2, and 3

Supplementary Table S4. Evaluation of the 102 differentially hydroxymethylated regions among dizygotic twin pairs discordant for cardiovascular death using the binary linear classifier

	N	Sensitivity	Specificity	Positive predictive value	Negative predictive value	Accuracy
**CVD-dDZ**	19 pairs	89.5%	63.2%	70.8%	85.7%	76.3%
**CVD-dMZ**	19 pairs	68.4%	52.6%	59.1%	62.5%	60.5%
**CVD-dDZ and CVD-dMZ combined**	38 pairs	78.9%	57.9%	65.2%	73.3%	68.4%

Abbreviations: CVD=cardiovascular death; CVD-dMZ= monozygotic twin pairs discordant for cardiovascular death; CVD-dDZ= dizygotic twin pairs discordant for cardiovascular death

Supplementary Figure S1: Up to top five biological functions and pathways from bioinformatic analysis of genes mapped to differentially hydroxymethylated regions (DhMRs) with Enrichr [1]

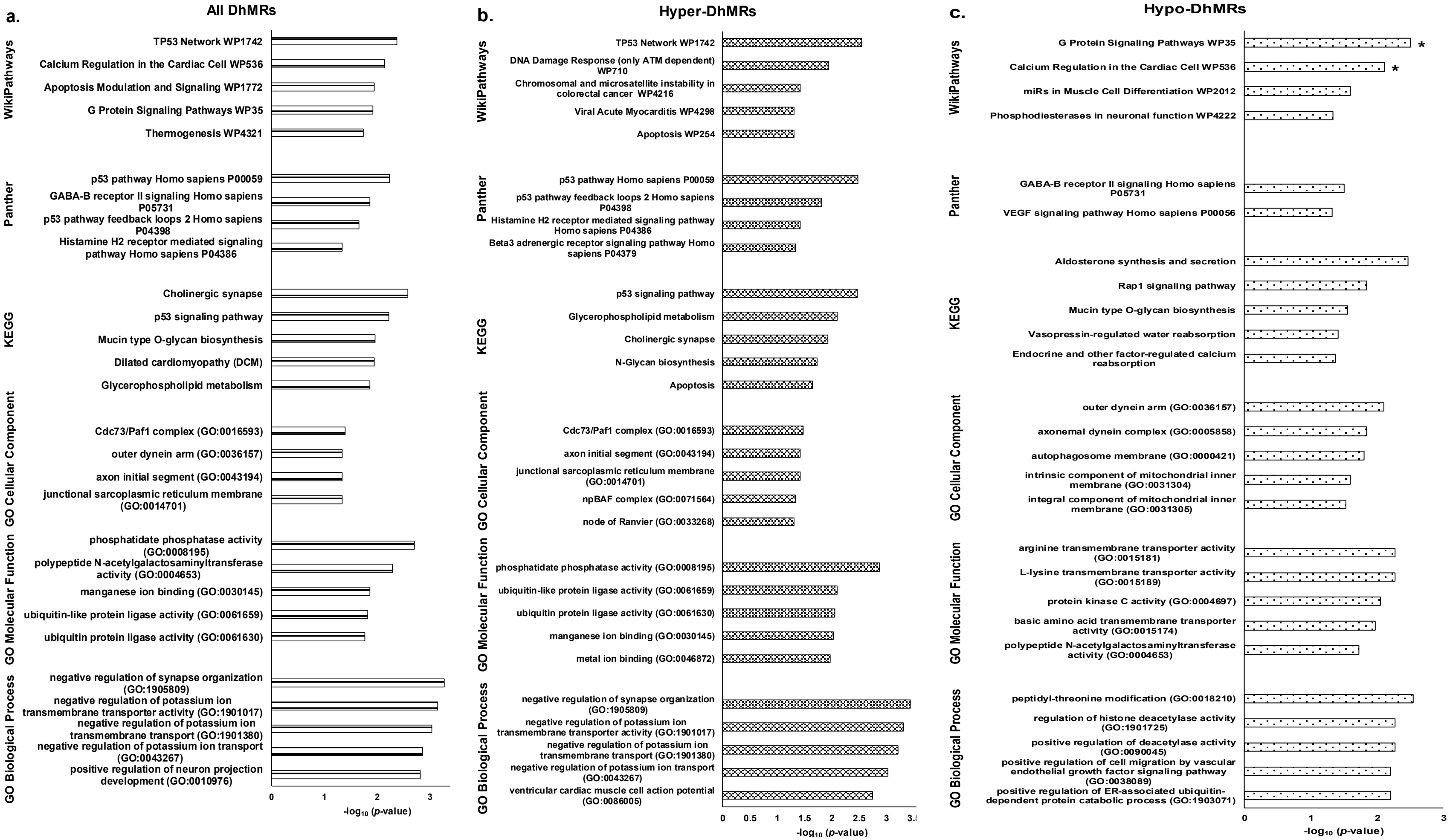

[1] up to top 5 enriched terms with the smallest nominal *p*-values<0.05. *$p_{adj}<0.05$, multiple-testing adjusted *p*-value using the Benjamini-Hochberg False Discovery Rate (FDR) procedure

DhMRs= differentially hydroxymethylated regions; hyper-DhMRs= differentially hyper-hydroxymethylated regions (case vs. control twins); hypo-DhMRs= differentially hypo-hydroxymethylated regions (case vs. control twins)

Supplementary Figure S2: Up to top five phenotypes, disorders, and diseases from bioinformatic analysis of genes mapped to differentially hydroxymethylated regions (DhMRs) with Enrichr [1]

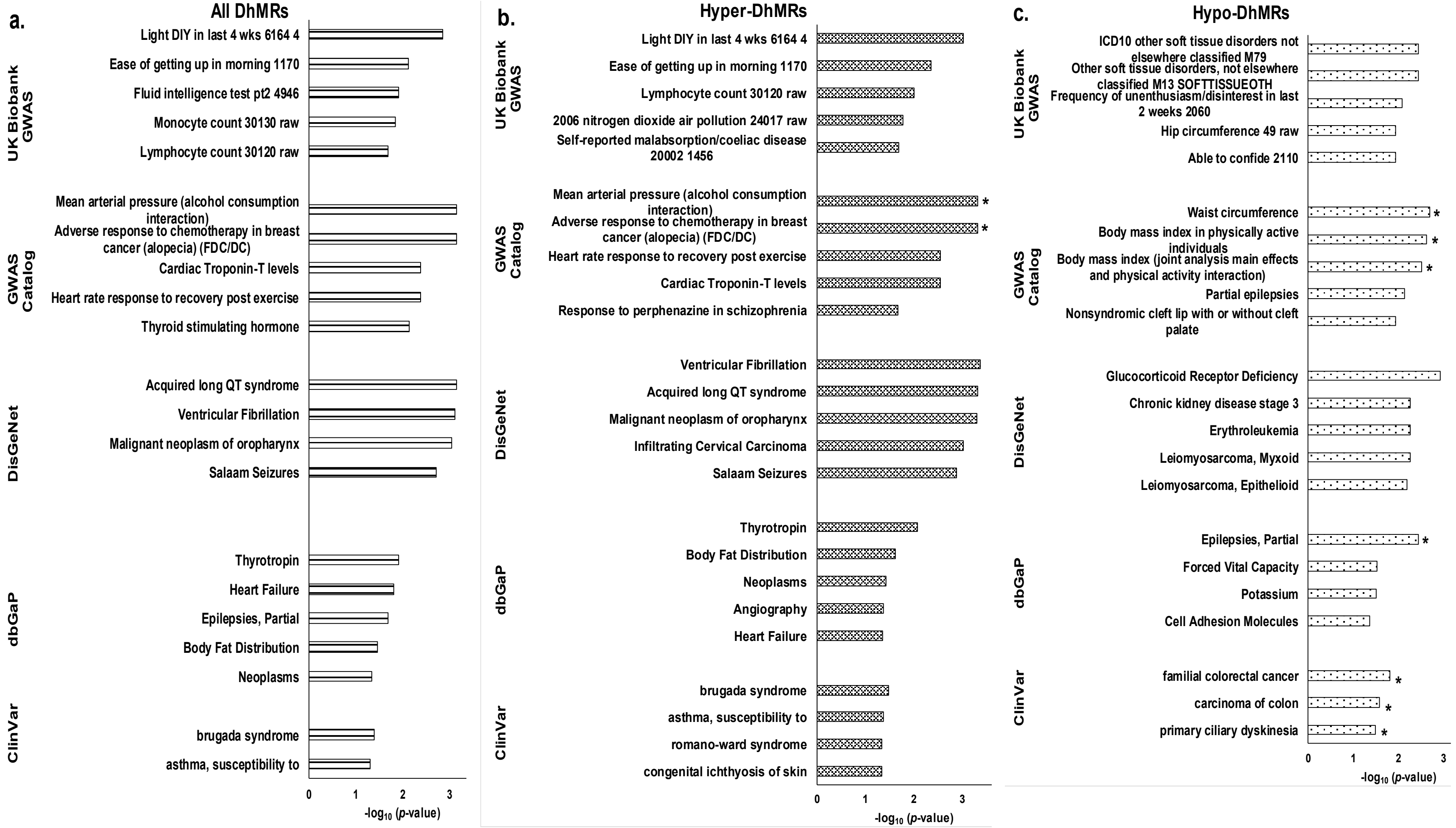

[1] up to top 5 enriched terms with the smallest nominal p-values<0.05. *$p_{adj}<0.05$, multiple-testing adjusted p-value using the Benjamini-Hochberg False Discovery Rate (FDR) procedure

DhMRs= differentially hydroxymethylated regions; hyper-DhMRs= differentially hyper-hydroxymethylated regions (case vs. control twins); hypo-DhMRs= differentially hypo-hydroxymethylated regions (case vs. control twins)

Supplementary Figure S3: Up to top five rare diseases/disorders from bioinformatic analysis of genes mapped to differentially hydroxymethylated regions (DhMRs) with Enrichr[1]

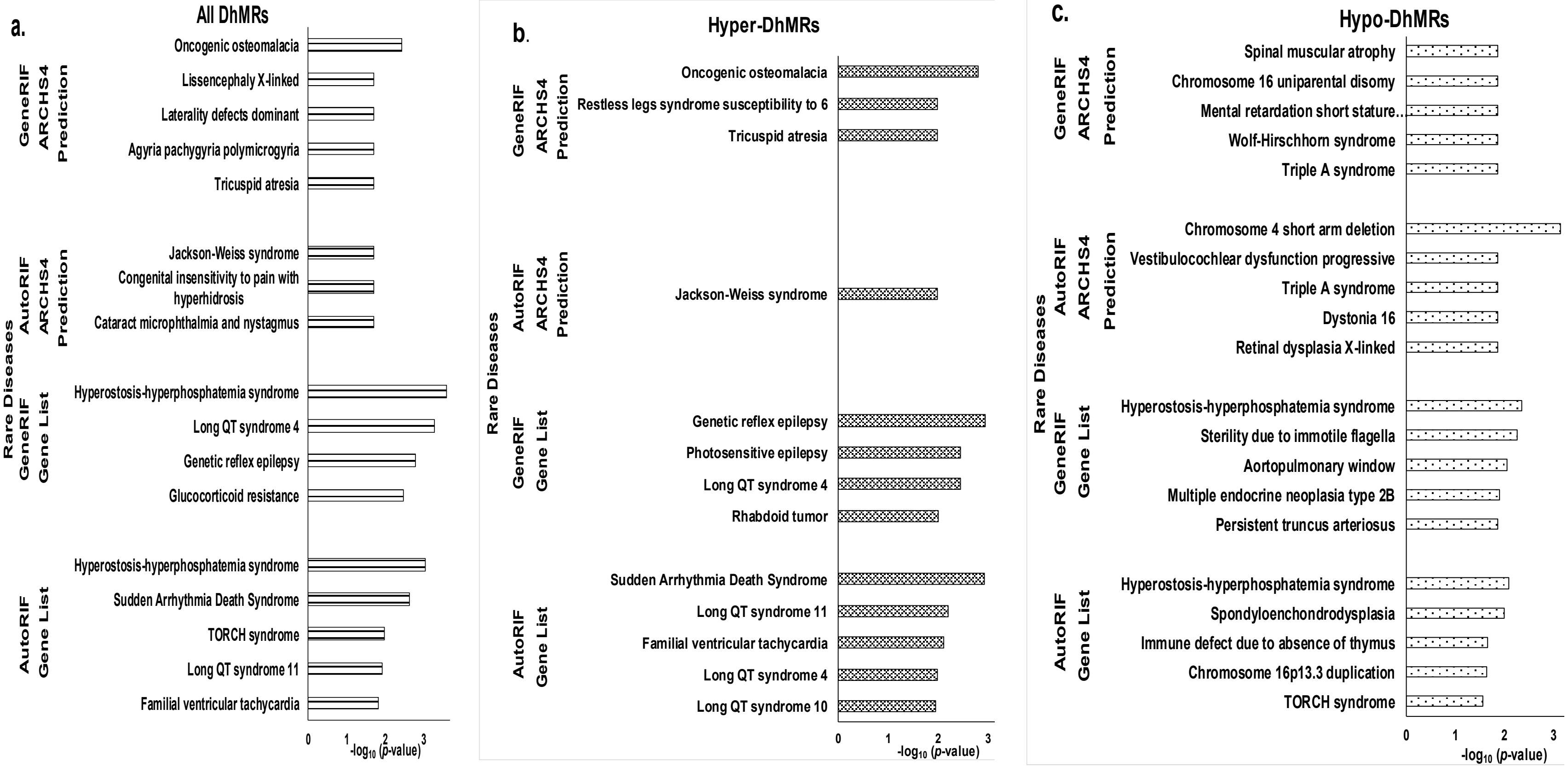

[1] up to top 5 enriched terms with the smallest nominal *p*-values<0.05.

DhMRs= differentially hydroxymethylated regions; hyper-DhMRs= differentially hyper-hydroxymethylated regions (case vs. control twins); hypo-DhMRs= differentially hypo-hydroxymethylated regions (case vs. control twins)
